# 达沙替尼单药治疗慢性髓性白血病髓内T淋巴细胞急变1例

**DOI:** 10.3760/cma.j.cn121090-20240110-00016

**Published:** 2024-06

**Authors:** 艳 张, 建斌 杨, 艳梅 马

**Affiliations:** 长治医学院附属和济医院血液肿瘤科，长治 046000 Department of Hematology and Oncology, Heji Hospital Affiliated to Changzhi Medical College, Changzhi 046000, China

患者，男，20岁。2021年3月初发现颈部数枚黄豆大小淋巴结，无触痛及发热，查血常规：WBC 25.8×10^9^/L，中性粒细胞11.27×10^9^/L，RBC 4.94×10^12^/L，HGB 139 g/L、PLT 178×10^9^/L，未治疗。颈部淋巴结进行性肿大，2021年4月底就诊于当地医院查血常规：WBC 101.8×10^9^/L，RBC 4.44×10^12^/L，HGB 125 g/L，PLT 127×10^9^/L，嗜酸性粒细胞1.14×10^9^/L，嗜碱性粒细胞0.15×10^9^/L。颈部超声：颈部双侧可见多个低回声结节，界清，最大约5.1 cm×3.5 cm，内结构异常，CDFI内可见血流信号。骨髓象：增生明显活跃，粒∶红为14.0∶1；粒系早幼粒细胞占5％，余以中性中晚幼粒细胞以下阶段为主，中性粒细胞胞质内颗粒缺乏特异性或颗粒分布不均匀，易见核固缩，中性分叶核粒细胞可见P-H畸形、可见核出芽；红系比例明显低，以中晚幼红细胞为主，成熟红细胞大小不等；原始单核细胞占18.0％，该类细胞胞体大小不一，胞质量较多，色灰蓝，部分核形态不规整，染色质较细，核仁尚清（1～2个）；全片共见巨核细胞87个，分类25个，颗粒型23个，产板型2个，血小板可见成堆。血象：粒系早幼粒细胞以下各阶段均可见，中性粒细胞易见核固缩、P-H畸形；成熟红细胞大小不等；单核细胞比例极度增高，原始单核细胞占4％，成熟较差单核细胞占34％；血小板成簇、堆。过氧化物酶（POX）染色：11％弱阳性，89％阴性。患者因经济原因放弃进一步诊治。

2021年8月13日患者因意识障碍就诊我科，查体：烦躁不安，贫血貌，颈部双侧、锁骨上下、腋下、腹股沟触及多枚肿大淋巴结，最大约3 cm×4 cm，质韧，活动可，部分融合，无触痛。双侧扁桃体Ⅲ度肿大，胸骨下段压痛阳性，左肺可闻及湿啰音，肝肋缘下未触及，脾肋缘下约4 cm，病理征阴性。血常规：WBC 18.04×10^9^/L，中性粒细胞8.78×10^9^/L，淋巴细胞5.71×10^9^/L，RBC 0.63×10^12^/L，HGB 19 g/L，PLT 255×10^9^/L。上腹部超声：脾大（肋间厚5.0 cm）。颈胸腹联合CT：颈部双侧、颌下、锁骨上下、腋下、腹股沟、纵隔、腹腔及腹膜后多发肿大淋巴结（提示淋巴瘤）。患者极重度贫血，予输血改善缺血缺氧的症状，后完善相关检查。骨髓象：增生明显活跃，其中粒系占66.0％，红系占0.5％；粒系比例偏高，粒系成熟障碍，颗粒分布不均匀，空泡，核质发育不平衡，核染色质疏松；红系缺如，成熟红细胞大小不一；原始细胞占9％；环片一周见到巨核细胞93个，血小板成簇。血象：分类单核细胞相对偏高，原始细胞占6％，偶见幼稚粒细胞；成熟红细胞大小不一；血小板散在、成簇；POX染色：60％弱阳性，40％阴性。骨髓流式细胞术免疫表型：原始细胞分布区域可见异常细胞群体，约占有核细胞的7.3％，表达CD7、CD38、cCD3，部分细胞表达CD5，少数细胞表达CD2。提示异常的T淋巴细胞。骨髓活检：送检穿刺骨髓组织，造血组织容量45VOL％（造血组织45％，脂肪组织55％），呈骨髓增生活跃，造血组织粒、红系增生；粒系前体细胞可见，中、晚阶段细胞散在或成堆可见；红系原、早阶段细胞可见，以中晚阶段细胞为主，散在或小堆可见；巨核细胞2～4个/HPF，为多叶核；淋巴细胞散在可见，偶见小堆，浆细胞散在可见。Gomori染色：MF-0级。Fe染色：阴性。免疫组化：CD3、CD5（少量+），TdT（少量+），CD20、PAX-5、CD34（个别+），MPO粒系（+），CD235a红系（+），CD138（个别+）。诊断意见：造血组织增生活跃，原始T淋巴细胞比例增高（约10％）。骨髓细胞遗传学：骨髓染色体核型：46, XY, t（9; 22）（q34; q11）[16]/46, XY[4]。骨髓BCR::ABL1融合基因定性检测：P190型阳性，P210型、P230型阴性。颈部淋巴结活检组织病理检查：被膜增厚，镜下见淋巴细胞弥漫性增生，该细胞小至中等大小，胞质窄，核染色质颗粒状，分布不均，部分可见小核仁，核分裂象易见，间质小血管增生，淋巴结结构破坏，残留个别淋巴滤泡，边缘窦及髓质窦结构消失，肿瘤细胞浸润被膜。诊断：非霍奇金淋巴瘤，考虑为T淋巴母细胞性白血病/淋巴瘤。免疫组织化学示：CD2（部分+），CD3（+），CD5（+），CD7（+），TdT（+），CD99（+），Ki67（30％+），CD4（部分+），CD8（部分+），CD10（部分+），CD20（−）、Pax-5（−），CD21、CD23（个别残留FDC+），CD68（−），CD138（−），BcL-2（+），Bcl-6（−），CD56（−）。EBER原位杂交（−）。淋巴结FISH检查（图1）：BCR::ABL融合基因阳性（检测位点为22q11.2/9q34）。淋巴结BCR::ABL融合基因实时定量PCR检测：BCR::ABL融合基因P190型阳性，BCR::ABL1/ABL1为109.802％。

**图1:**
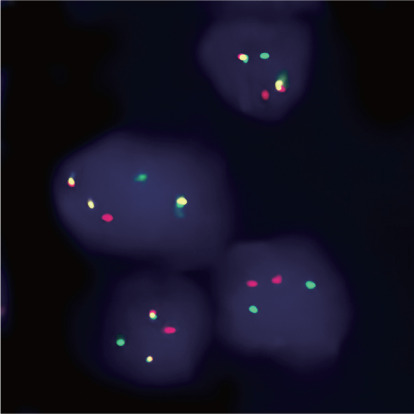
该例慢性髓性白血病患者淋巴结FISH检查

根据患者症状、体征及辅助检查结果，最终确诊慢性髓性白血病（CML）急变期（髓内T淋巴细胞急变伴髓外累及）。患者于2021年9月3日开始口服达沙替尼100 mg/d，用药4 d后出现双侧睾丸鞘膜腔微量积液，考虑与用药有关，经对症治疗后好转。2021年10月8日（达沙替尼治疗第36天）血常规：WBC 4.7×10^9^/L，RBC 3.63×10^12^/L，HGB 114 g/L，PLT 163×10^9^/L，淋巴细胞3.13×10^9^/L，提示获完全血液学缓解。2022年1月4日（达沙替尼治疗4个月）复查CT提示淋巴结明显缩小伴部分消失。骨髓象：原始细胞占6.5％。骨髓BCR::ABL1/ABL1为1.03％，提示获主要分子学缓解。患者因经济原因拒绝移植，遂继续口服达沙替尼维持治疗。2022年9月2日（达沙替尼治疗1年）复查骨髓染色体核型分析未见异常，提示获完全细胞遗传学缓解。动态监测BCR::ABL1融合基因（P190型）结果显示患者近两年BCR::ABL1/ABL1均小于1％。2024年1月31日复查骨髓未见原始细胞。

